# A Concise Review and Required Precautions for COVID-19 Outbreak in Diagnostic and Interventional Radiology

**DOI:** 10.1155/2020/7159091

**Published:** 2020-07-22

**Authors:** Ali Kord, Behnam Rabiee, Siwen Wang, Sara Rostami, Ron C. Gaba, Karen L. Xie

**Affiliations:** ^1^Department of Radiology, University of Illinois at Chicago, Chicago, IL, USA; ^2^Division of Interventional Radiology, University of Illinois at Chicago, Chicago, IL, USA; ^3^Biomedical Visualization Graduate Program, University of Illinois at Chicago, Chicago, IL, USA

## Abstract

A global outbreak of a novel coronavirus (COVID-19) pneumonia began in December 2019 in Wuhan, China. The World Health Organization (WHO) announced a pandemic on March 11, 2020. The rapid rise in the case numbers and mortality led to the saturation of hospitals in many countries. COVID-19 patients usually present with fever, fatigue, dry cough, and dyspnea. Given the shortage of diagnostic kits in many countries and very high sensitivity of computed tomography (CT) for diagnosis of COVID-19 in clinically suspicious patients, the chest CT has been implemented among the primary initial methods of diagnosis before the confirmatory laboratory tests. This puts radiologists and radiology staff on the front line of this alarming pandemic. This report summarizes chest CT findings of COVID-19 patients to facilitate diagnosis and reviews a list of necessary precautions and safety measures for diagnostic and interventional radiology personnel. These precautionary plans are extremely important to avoid contamination of the health-care providers, as well as cross-contamination between patients.

## 1. Introduction

Beginning on December 31, 2019, the World Health Organization (WHO) was informed of a cluster of cases of pneumonia with unknown causes reported in Wuhan, Hubei province, China [1]. The patients presented with progressive pneumonia symptoms including fever, dry cough, fatigue, and respiratory distress.

A novel coronavirus, severe acute respiratory syndrome coronavirus 2 (SARS-CoV-2, previously known as 2019-nCoV), was isolated from the respiratory tract secretions of the patients by January 7, 2020 [2–4], and the disease was named as coronavirus disease 2019 (COVID-19) soon thereafter.

## 2. What Is Known About COVID-19

Being a member of the Coronaviridae family, the novel coronavirus (2019-nCoV) is a nonsegmented, enveloped, positive-sense, single-strand ribonucleic acid virus [5, 6]. This virus has a significant capability to infect humans through an interaction between the viral S protein and the angiotensin-converting enzyme 2 receptors on human respiratory endothelial cells [3]. It spread rapidly inside China and transmitted to other countries [4, 7]. As early as 45 days after the first notice, more than 60,000 cases were reported worldwide, with 1,525 mortalities, providing the preliminary estimation of a 2% mortality rate [4, 8–11]. By March 11, 2020, WHO officially announced COVID-19 as a pandemic, declaring a worldwide health emergency state [12]. As of this report on May 14, 2020, the number of cases worldwide has reached 4,258,666 in more than 216 countries with 294,190 deaths, which estimates the mortality rate of 6.9% [11]. These numbers highlight the significant virulence of the novel coronavirus.

Two previous outbreaks of coronavirus, severe acute respiratory syndrome (SARS) and Middle East respiratory syndrome (MERS) coronavirus, which had high mortality of 11% and 30%, respectively, have alarmed the medical community to the potential of these respiratory infections [5, 13]. Despite their high mortality, comparing the number of cases and countries affected by the epidemics demonstrates that the transmission rate of those viruses was significantly lower than that of the novel coronavirus (2019-nCoV); MERS affected a total of 2,500 cases in 27 countries, and SARS infected 8,422 cases in 29 countries [5, 14]. This highlights the increased risks of disease exposure and transmission to front-line health-care professionals in treating the patients and the importance of taking necessary precautions and safety measures to protect the health-care personnel during the current pandemic [15, 16].

## 3. Radiology Personnel Are among the Front-Line Caregivers for COVID-19

The diagnostic gold standard for viral infections is usually a real-time polymerase chain reaction (RT-PCR) of the respiratory tract specimens. In general, nasopharyngeal or throat swabs are used for viral diagnostics. Sputum and feces specimens can certainly add to the diagnostic yield if nasopharyngeal or throat swabs are negative for COVID-19. Furthermore, although bronchoalveolar lavage is a very relevant specimen, it may not be always possible in severely ill patients. In COVID-19 cases, the routine RT-PCR from nasopharyngeal swaps has a significantly limited sensitivity of 60–70%, especially in the early days of infection [17, 18], which may be due to sample error and the choice of specimen. On the other hand, chest computed tomography (CT) has been investigated as a tool to primarily diagnose the patients with COVID-19 pneumonia, which showed a high sensitivity of 97% in selected patients, even before the PCR test read positive [18]. Therefore, in many countries, a chest CT has been implemented among the primary methods of diagnosis, although sufficient data are lacking. This puts radiologists and radiology staff on the front line of this alarming pandemic. Hence, taking the necessary precautions is deemed to be essential to protect the radiology personnel, while maintaining their efficiency to provide service to the patients.

**Figure 1:**
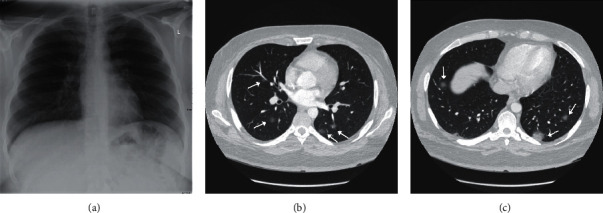
A 26-year-old male with a recent diagnosis of diabetes presented with several days of progressive myalgia, cough, chills, and dyspnea and intermittent generalized crampy mild abdominal pain. (a) The chest radiograph was negative for consolidation. (b, c) The CT pulmonary arteriogram was performed for the same patient to rule out pulmonary embolism and showed multiple patchy, lower-lobe-prominent, nodular ground-glass opacities (arrows). The patient was tested positive for COVID-19.

**Figure 2:**
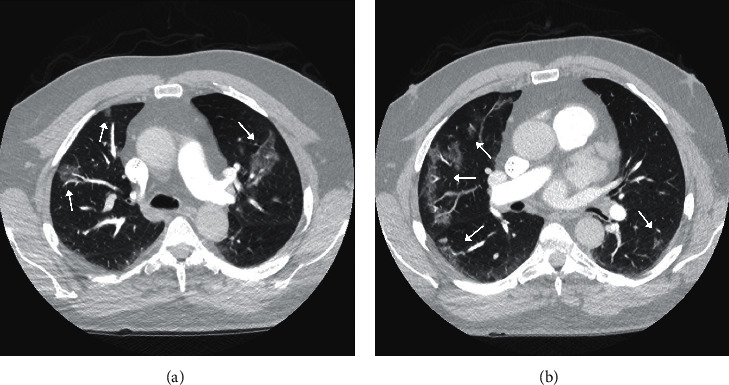
A 67-year-old male with a past medical history of sudden cardiac arrest with the placement of a defibrillator and a recent travel history to Europe presented with nonproductive cough, myalgia, and fever. (a, b) The CT pulmonary arteriogram was performed to rule out pulmonary embolism and demonstrated multiple patchy, peripheral, more organized ground-glass opacities (arrows) compared to the patient in Figure 1. The patient was tested positive for COVID-19.

Radiologic findings of COVID-19 patients can be nonspecific and overlap with pneumonia caused by other viruses. The chest radiograph may be not sensitive enough to detect early disease changes (Figure 1). Typical CT features are described as bilateral multiple lobular and subsegmental areas of consolidation in patients admitted in the intensive care unit (ICU) and bilateral ground-glass opacity and subsegmental areas of consolidation in non-ICU patients (Figure 1) [14]. The abnormalities are typically in peripheral and lower-lobe distributions (Figure 2). Another study indicated that the findings could be unilateral in up to 25% of the cases [19]. In resolving cases with less severe disease, later, chest CT images showed bilateral ground-glass opacities, whereas the consolidation had resolved [13]. In more severe cases leading to death, serial chest CT showed progressively worsening bilateral consolidation during a course of 7 days [5]. Overall, the radiologic findings are similar to those of MERS and SARS [13] and can also overlap with atypical pneumonia, cytomegalovirus pulmonary involvement, as well as H1N1 influenza. Specifically, in the high risk groups with prior pulmonary diseases, CT findings may be less specific. Therefore, gathering a relevant history and physical examination could help raise the alarm for COVID-19 in such patients with the aforementioned chest CT findings.

## 4. How to Stay Safe while Providing Critical Patient Care

The novel coronavirus (2019-nCoV) is highly contagious, and the main route of transmission is believed to be respiratory droplets. The virus can stay active for several hours to days on multiple surfaces under artificial conditions, and touching the face or the mucosal surfaces of the body with contaminated hands could probably lead to infection. While the highest risk of transmission via droplets is within 3 feet (91.44 cm) of the source, they can contaminate up to 6 feet (183 cm) [5, 20].

**Figure 3:**
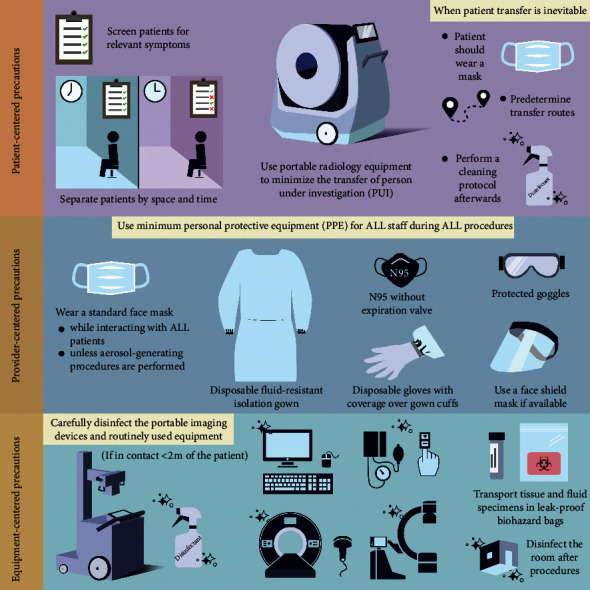
Proposed precautions for diagnostic radiology for COVID-19 outbreak based on the WHO and CDC recommendations.

According to the latest WHO and Center for Disease Control and Prevention (CDC) guidelines and considering the ongoing research on the subject, the necessary precautionary recommendations fall under three main categories: patient-centered precautions, provider-centered precautions, and equipment-centered precautions (Figure 3). We added an additional category for Interventional Radiology- (IR-) specific precautions. Some of the patient-centered and provider-centered precautions may also be useful and overlap with the operation room precautions.

### 4.1. Patient-Centered Precautions

All patients should be screened for relevant symptoms. Patients who meet the criteria for a person under investigation (PUI) require isolation and additional precautions to provide triage and care.

To minimize the risk of cross-infection, patients with different infection risks should be separated by space where possible or by time otherwise [21]. Additionally, procedures on PUI should be separated by place and time from other patients [21].

The transfer of PUIs should be kept at a minimum. Utilizing portable radiology equipment in this setting proved to be a very effective safety measure in the SARS epidemic [5, 13, 22, 23]. For complicated cases in which patient transfer is inevitable, assigning dedicated procedural rooms and predetermined transport routes and performing a cleaning protocol according to infectious disease control protocol afterward will greatly minimize the risk of cross-contamination. It is important that the COVID-19 patients wear a surgical mask during the transfer and, if possible, during the procedures which require direct provider contact.

### 4.2. Provider-Centered Precautions

Based on the experience of the SARS epidemic, it is clear that the risk of virus transmission to the providers is significantly reduced by using droplet and contact precautions [13, 23–25]. The current evidence suggests asymptomatic transmission even through the incubation period [26]. This highlights the importance of the use of minimum personal protective equipment (PPE) for all staff during all procedures, preferably everywhere or, at least, at the involved centers.

The latest recommendations of WHO include respiratory protection with a standard face mask for the health-care providers while interacting with all patients, unless aerosol-generating procedures are performed [5, 27, 28]. Additionally, CDC recommends strict airborne precaution using an N95 mask or higher when in close contact with a confirmed COVID-19 case, or a PUI [5]. Furthermore, necessary PPE includes a disposable (if resources are limited, autoclavable) fluid-resistant isolation gown, disposable gloves with the emphasis of coverage over gown cuffs, protective goggles, and, if available, a face shield [29]. Recent studies also reported the possibility of transmission via ocular surface mucosa, which highlights the importance of ocular protection wear such as glasses or face shields as well [30, 31].

Rapid spread of COVID-19 pandemic highlights the importance of the awareness and effective measures to prevent transmission of microorganisms, particularly highly resistant microorganisms. Therefore, it is important to be mindful of general infection prevention protocols, such as hand hygiene for everyone including patients and health-care providers. In addition, lessons learned from the present outbreak management of COVID-19 call for a continuing quality improvement program in place for the infection prevention and control to prepare for potential outbreaks in the future.

### 4.3. Equipment-Centered Precautions

The use of portable imaging devices is highly recommended specifically for the confirmed COVID-19 patients, as well as the PUI. These devices should be carefully disinfected before and after the procedures. All the routinely used equipment including CT and magnetic resonance imaging (MRI) machines, ultrasound transducers, blood pressure monitoring cuffs, pulse oximeters, and reading room mice, keyboard, monitors, and surfaces, as well as interventional radiology suite equipment, should be disinfected following every COVID-19 suspect encounter, if in contact <2 m of the patient, which should be avoided whenever possible [5].

CDC recommendations include washing with soap and water on the possible surfaces and equipment or alternatively use of at least intermediate-level disinfectants such as ethyl alcohol, isopropyl alcohol, and iodophor germicidal detergent solution [5]. It is important to note that ethanol solutions should contain at least 70% ethanol for surfaces and at least 60% ethanol for hand sanitizers, according to CDC guidelines [32]. Education of the staff on the decontamination protocols plays an important role to prevent cross-contaminations in these settings [33].

Tissue and fluid specimens are considered infectious and must be transported in leak-proof biohazard bags by trained personnel to ensure safe handling practices and spill decontamination procedures [21]. Disinfection of the room and medical equipment must be performed after procedures on these patients as well [21].

### 4.4. Interventional Radiology-Specific Precautions

**Figure 4:**
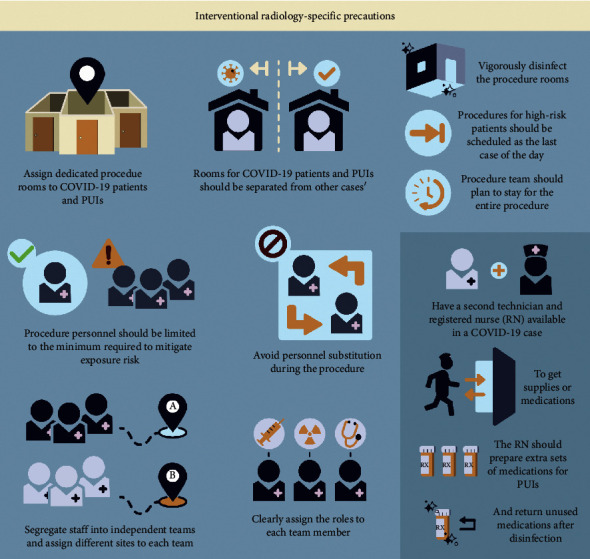
Proposed precautions for interventional radiology for COVID-19 outbreak based on the WHO and CDC recommendations and ongoing studies.

Specific precautions should be taken for interventional radiology suites to lower the risk of disease spread [34]. While the interventional radiology team may continue with urgent (e.g., oncologic and dialysis management procedures) and emergent (e.g., abscess drainage) procedures, elective cases should be deferred in the interest of resource conservation and exposure risk mitigation. To this end, it may be useful to generate a stratification system for procedure urgency to guide scheduling; a proposed protocol may be to assign procedures color codes to indicate urgency (red = schedule within 2 weeks, orange = schedule within 2–4 weeks, yellow = schedule within 4–8 weeks, and green = schedule after 8 weeks). Regardless of the system used, scheduling alterations require more targeted patient triage and communication of measures taken to protect patients [21]. A proposed protocol for the COVID-19 outbreak, adapted from our institutional protocol, has been summarized in the following paragraphs and Figure 4. Regarding outpatient clinic visits, these can be performed as telehealth visits using video or phone conferencing.

While portable procedure performance is preferred, specific and dedicated procedure rooms should be dedicated to the PUIs or confirmed COVID-19 patients, and these rooms should be separated from other procedure rooms [23]. The procedure rooms should be vigorously disinfected following each procedure on a suspected patient. If possible, performing the procedure on COVID-19 patients or PUIs should be scheduled as the last case of the day to minimize the risk of cross-contamination.

Procedure personnel should be limited to the minimum required for safe and effective performance, in order to minimize exposure risk to the personnel. The procedure team should plan to stay for the entire procedure from start to finish (i.e., no breaks or substitute personnel). Medical doctor coverage should be limited to a single person in order to keep the risk of infection and losing manpower to a minimum unless case complexity requires multiple doctors. No nonessential personnel (e.g., medical students) should scrub into cases during the COVID-19 outbreak. For medical teams covering multiple hospitals, assigning different sites to each subteam will minimize the cross-contamination risk [21, 23]. In the case of intrahospital transmission, segregation of staff into independent teams will be needed to prevent a shutdown of the entire service should there be a need for quarantine [21, 23]. The assigned role of each member of the provider team should be clear to minimize confusion during the probable overflow hours, which will require more detailed protocols than usual.

It is helpful to have a second technician and registered nurse (RN) available to get supplies or medications for a COVID-19 case to prevent procedural personnel from leaving the room during the procedure. This may include assigning a second on-call technician for the same reason and particularly for more complicated cases. The RN should predict and prepare extra sets of medications for the suspicious case, and unused medications may be returned after the conclusion of each procedure following disinfection protocol.

## 5. Conclusions

With the rising number of COVID-19 cases all over the world, including throughout the United States, radiology departments are expected to confront a more than usual volume of critical patients. Radiology departments and the IR team should be in close communication with their affiliated hospital units and providers. Strict adherence to WHO and CDC policies and guidelines is the main primary defense against spreading the infection among health-care workers. These include measurements such as hand hygiene, appropriate use of PPE, disinfecting equipment and procedure rooms, and educating the personnel on the most current policies and recommendations.
